# Use of indocyanine green fluorescence versus patent blue V dye for sentinel lymph node biopsy in early breast cancer, a randomized controlled trial

**DOI:** 10.1186/s43046-026-00403-5

**Published:** 2026-09-02

**Authors:** Muhammad Nida, Ashraf Abdelmoghny, Ahmed G. Osman, Mohammad A. Abd-erRazik, Dina M. Hanafy

**Affiliations:** 1 Department of Surgical Oncology, Tanta Cancer Center, Tanta, Egypt; 2 Department of General Surgery, Faculty of Medicine, Ain Shams University, Cairo, Egypt

**Keywords:** Breast Cancer, Sentinel Lymph Node, Indocyanine Green Fluorescence, Patent Blue V

## Abstract

**Background:**

Sentinel lymph node biopsy (SLNB) is standard for axillary staging in early breast cancer. While the combination of radioisotope and blue dye (e.g., patent blue V, PBV) remains the standard, it has limitations including logistics, variable identification rate (IR), and allergic potential. Indocyanine green (ICG) fluorescence is a promising alternative, but high-quality comparative evidence is needed.

**Methods:**

This was a single-center, prospective, randomized controlled trial. Forty patients with early-stage, node-negative breast cancer were allocated to SLNB using either ICG (*n* = 20) or PBV (*n* = 20). All patients subsequently underwent completion level I–II axillary lymph node dissection (ALND) as the pathological reference standard for diagnostic performance assessment. Primary outcome was sentinel lymph node (SLN) IR. Secondary outcomes included detection time, number of SLNs retrieved, false-negative rate (FNR), and safety.

**Results:**

Baseline characteristics were comparable between groups. The SLN IR was significantly higher with ICG (100% [20/20]) than with PBV (75% [15/20], *p* = 0.047). ICG was associated with a significantly shorter median detection time (14.5 vs. 24.0 min, *p* < 0.001) and retrieved more SLNs (mean: 3.6 vs. 2.4, *p* = 0.002). Most critically, ICG demonstrated 100% sensitivity, specificity, negative predictive value (NPV), and overall diagnostic accuracy, with a 0% FNR. In contrast, PBV achieved a sensitivity of 75%, an overall diagnostic accuracy of 90%, and an FNR of 25%. No ICG-related adverse events occurred. PBV caused skin discoloration in 75% of patients and one (5%) allergic reaction.

**Conclusion:**

ICG fluorescence achieved a higher SLN IR, shorter detection time, higher sensitivity, lower FNR, and fewer tracer-related adverse events than PBV as a single tracer for SLNB in patients with early-stage breast cancer. These findings suggest that ICG is a promising standalone tracer when radioisotope mapping is unavailable. Larger multicenter studies are required before widespread adoption can be recommended.

## Introduction

Breast cancer remains the most frequently diagnosed cancer globally [1, 2], with axillary lymph node (LN) status being a paramount prognostic factor and a critical determinant of adjuvant treatment strategies. Sentinel lymph node biopsy (SLNB) has replaced axillary lymph node dissection (ALND) as the standard of care for staging the clinically node-negative axilla, significantly reducing the morbidity associated with surgery while providing essential prognostic information [3]. Landmark randomized trials, including the Milan [4, 5] and NSABP B-32 [6–8] trials, established the oncologic safety of SLNB in node-negative patients. Subsequently, the ACOSOG Z0011 [9–11] and the more recent SENOMAC trial [12] demonstrated that completion ALND can be safely omitted in selected patients with one or two positive sentinel lymph nodes (SLNs), while the AMAROS [13, 14] and OTOASOR [15] trials showed that axillary radiotherapy provides regional control comparable to ALND with lower treatment-related morbidity. More recently, the SOUND [16] and INSEMA [17] trials have suggested that even SLNB may be safely omitted in carefully selected patients.

Despite this progressive de-escalation of axillary surgery, accurate identification of the SLN remains fundamental to accurate axillary staging whenever SLNB is indicated. The dual-tracer approach, combining a radioisotope (technetium-99m) and a blue dye (e.g., patent blue V - PBV), is considered the reference standard [18]. However, radioisotope mapping is unavailable in many regions because of logistical, regulatory, and financial limitations. Consequently, in many institutions, particularly those without access to nuclear medicine facilities, blue dye alone continues to be used despite its variable identification rate (IR) (often cited between 85 and 95%), risk of allergic reactions including rare but life-threatening anaphylaxis, and frequent, transient skin discoloration that patients can find distressing [19, 20].

Indocyanine green (ICG) fluorescence imaging has emerged as a promising alternative tracer. ICG has a long-established safety profile in other medical domains, including cardiac output measurement, hepatic function assessment, ophthalmic angiography [21], and hepatobiliary surgery [21, 22]. It is a water-soluble compound that, when excited by near-infrared (NIR) light, emits fluorescence that can be detected by specialized camera systems. This technology allows for real-time, high-contrast visualization of lymphatic vessels and nodes [21]. Proposed advantages include superior tissue penetration compared to visible light, no need for radiation protocols, and an excellent safety profile with minimal risk of allergic reactions [23]. Multiple prospective studies and randomized trials have demonstrated high SLN IR with ICG, findings that were further strengthened by the recent multicenter randomized INFLUENCE Trial, which demonstrated that ICG alone was non-inferior to ICG combined with either radioisotope or blue dye [24]. While recent randomized and prospective studies have substantially strengthened the evidence supporting ICG as a standalone tracer [25], direct randomized comparisons between ICG and blue dye alone remain limited, particularly in resource-limited settings where radioisotope mapping is not routinely available and blue dye continues to be used as the sole tracer.

Reliable validation of novel SLNB mapping techniques requires accurate assessment of diagnostic performance, including false-negative rate (FNR) and sensitivity, against an appropriate pathological reference standard. Therefore, we conducted this prospective randomized controlled trial (RCT) to directly compare the efficacy and safety of ICG fluorescence versus PBV dye for SLNB in patients with early-stage breast cancer. Accordingly, we conducted this prospective randomized controlled trial to compare the efficacy and safety of ICG fluorescence and PBV as single tracers for SLNB in patients with early-stage breast cancer. We hypothesized that ICG would achieve a higher SLN IR, favorable diagnostic accuracy, and fewer adverse events compared to PBV.

## Methods

### Study design

This was a single-center, prospective, open-label, RCT conducted at the Breast Surgery Unit of Ain Shams University Hospitals.

### Participants

Female patients with treatment-naive early-stage breast cancer were assessed for eligibility. Inclusion criteria were: age between 18 and 70 years; histologically confirmed invasive breast carcinoma (T1–2 tumors ≤ 5 cm); and a clinically and radiologically negative axilla (cN0) confirmed by ultrasonography. Exclusion criteria were: clinical or radiological evidence of nodal (N1–3) or distant metastases (M1); any prior history of breast or axillary surgery, radiotherapy, or systemic therapy (chemotherapy, hormonal therapy, or targeted therapy); and known hypersensitivity to iodine, ICG, or PBV.

### Randomization and masking

Eligible patients were randomly allocated in a 1:1 ratio to either the ICG group or the PBV group. The randomization sequence was computer-generated using the online platform *random.org*, with variable block sizes to ensure allocation concealment. Assignments were sealed in sequentially numbered, opaque envelopes, which were opened by an operating room nurse after induction of anesthesia. Due to the nature of the interventions (visual vs. fluorescent detection), blinding of the surgeons was not feasible. Furthermore, pathologists were not blinded to group allocation because of the persistent blue discoloration of nodes in the PBV group. All histopathological diagnoses were made using standardized criteria to minimize interpretation bias.

### Interventions

- ICG Protocol (Intervention Group): Patients received a peri-areolar subdermal injection of 2 mL (5 mg total dose) of ICG (Verdye^®^, Diagnostic Green GmbH; 2.5 mg/mL concentration). The injection site was gently massaged for only 5 min according to the established operative protocol to facilitate lymphatic uptake while minimizing excessive lymphatic diffusion and background axillary fluorescence. An NIR fluorescence imaging system (IMAGE1 S™ with HOPKINS^®^ NIR/ICG scope and D-LIGHT P SCB light source, Karl Storz, Tuttlingen, Germany) was used to identify and subsequently excise all fluorescent SLNs.
- PBV Protocol (Control Group): Patients received a peri-areolar subdermal injection of 2 mL of PBV dye (Patent Blue V S.A.L.F. 2.5%, S.p.A. Laboratorio Farmacologico). The injection site was massaged for 15 min. The axilla was explored, and all blue-stained LNs and any blue-afferent lymphatic vessels were identified visually and excised as SLNs.

In both groups, completion level I and II ALND was performed immediately after SLNB. Completion ALND served solely as the pathological reference standard for evaluating the diagnostic performance of SLNB, allowing calculation of sensitivity, specificity, negative predictive value (NPV), overall diagnostic accuracy, and FNR.

All patients underwent regular postoperative follow-up to ensure patient safety and document early procedure-related complications. During the first 24 h after surgery, patients were closely monitored for postoperative complications and tracer-related adverse reactions. After discharge, patients attended scheduled outpatient follow-up visits for assessment of short-term postoperative outcomes.

### Outcomes

The primary outcome measure was the SLN IR, defined as the proportion of patients in whom at least one SLN was successfully identified and retrieved.

Secondary outcome measures included:

1. The number of SLNs identified per patient.
2. The time from injection to intraoperative visualization of the first SLN (detection time).
3. The diagnostic performance of SLNB (sensitivity, specificity, NPV, overall accuracy, and FNR), using the combined pathological status of the excised SLN(s), when identified, together with the completion level I–II ALND specimen as the pathological reference standard.
4. The rate of tracer-related adverse events (e.g., allergic reactions, skin discoloration).

### Pathological analysis

All resected SLNs and ALND specimens were submitted for standard pathological processing with formalin fixation and paraffin embedding. Serial sectioning and hematoxylin and eosin (H&E) staining were performed for all nodes. Immunohistochemistry was performed at the pathologist’s discretion for equivocal cases. A node was considered positive for metastasis if any cluster of malignant cells was identified, including isolated tumor cells.

### Diagnostic accuracy analysis

Diagnostic performance was evaluated using the combined pathological status of the excised SLN(s), when identified, together with the completion level I–II ALND specimen as the pathological reference standard.

Two complementary analyses were performed. First, an all-randomized-patient analysis included all 20 patients in each treatment group. In this analysis, PBV mapping failures were retained according to the final pathological reference standard; consequently, mapping failure in a patient with metastatic axillary disease contributed to the false-negative count, whereas mapping failure in a patient with node-negative final pathology contributed to the true-negative count. The resulting sensitivity, NPV, FNR, and overall accuracy therefore reflect this analytical approach.

Second, a sensitivity analysis was performed among patients with successful SLN mapping only, thereby separating technical mapping failure from a true-negative SLNB result. This analysis included 20 patients in the ICG group and 15 patients in the PBV group.

### Statistical analysis

Sample size was prospectively determined for a pilot RCT. A total of 40 patients (20 per group) were specified in the study protocol to provide preliminary estimates of the SLN IR, diagnostic performance, safety, and variability of the two mapping techniques, thereby informing the design and sample size calculation of a future adequately powered definitive RCT.

Statistical analysis was performed using IBM SPSS Statistics version 26.0. Normality of continuous data was assessed using the Shapiro–Wilk test. Normally distributed continuous variables were presented as mean ± standard deviation (SD) and were compared using the independent samples *t*-test. Non-normally distributed data were presented as median and interquartile range (IQR) and were compared using the Mann–Whitney U test. Categorical variables were presented as counts and percentages and were compared using the chi-square test or Fisher’s exact test, as appropriate. Diagnostic performance measures were calculated from 2 × 2 contingency tables. A two-sided *p*-value < 0.05 was considered statistically significant.

## Results

### Patient enrollment and baseline characteristics

**Fig. 1:**
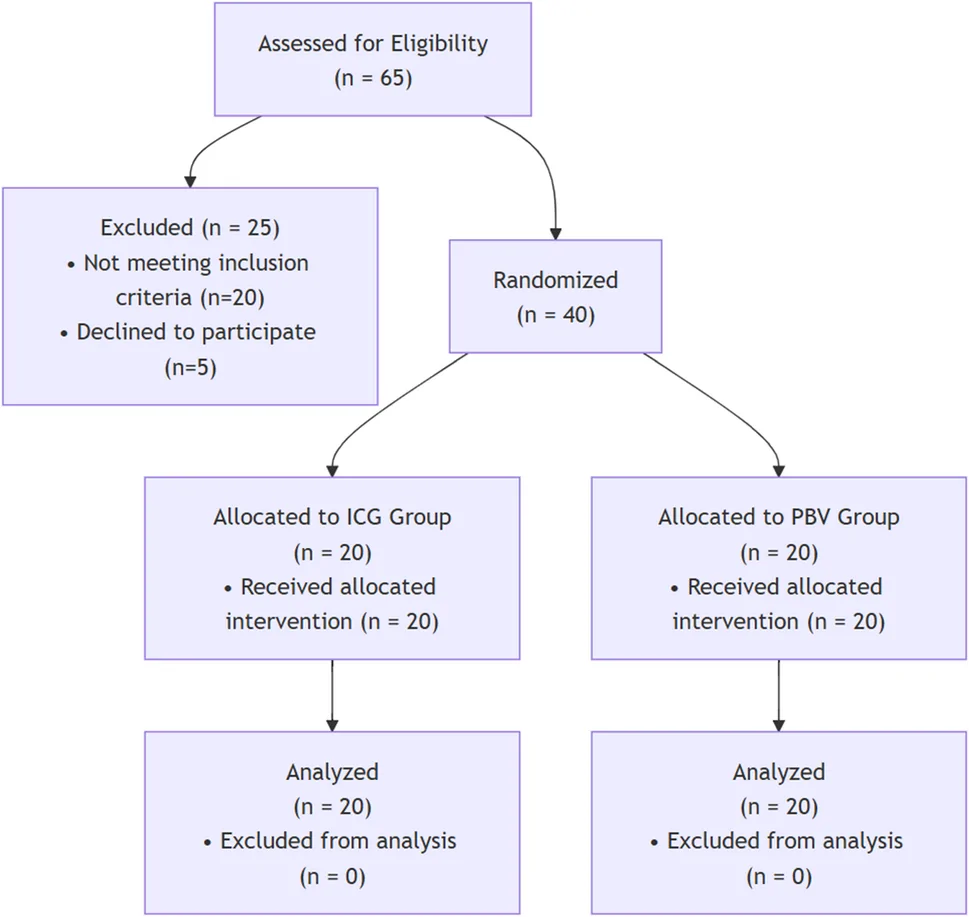
CONSORT flow diagram of the trial. This diagram illustrates the pathway of participants through the RCT, from assessment of eligibility through allocation, intervention, and final analysis. All enrolled patients (*n* = 40) received their allocated intervention (ICG fluorescence or PBV dye) and were included in the final intention-to-treat analysis

Between April 2023 and April 2025, 40 patients were enrolled and randomly assigned to either the ICG group (*n* = 20) or the PBV group (*n* = 20). All patients received their allocated intervention and were included in the final analysis (Fig. 1).

The two groups were well-matched at baseline, with no statistically significant differences in demographic or clinicopathological characteristics (Table 1). The mean age was 49.4 ± 10.9 years in the ICG group and 49.2 ± 11.7 years in the PBV group (*p* = 0.945). Comorbidities, including hypertension (25% in both groups) and diabetes mellitus (20% in both groups), were equally distributed. Body mass index was comparable between groups (29.7 ± 5.1 kg/m² vs. 26.5 ± 5.5 kg/m², *p* = 0.067). Tumor laterality, quadrant distribution, and clinical T-stage (T1: 45% vs. 45%; T2: 55% vs. 55%) were also similar (all *p* > 0.05).

**Table 1:** Baseline demographic and clinicopathological characteristics

Characteristic	ICG Group (*n* = 20)	PBV Group (*n* = 20)	*p*-value
Age, years			
Mean ± SD	*49.4 ± 10.9*	*49.2 ± 11.7*	*0.945*
Median (IQR)	*47.5 (40.5–57.5)*	*48.5 (40.5–57.5)*	
Body Mass Index, kg/m²			
Mean ± SD	*29.7 ± 5.1*	*26.5 ± 5.5*	*0.067*
Median (IQR)	*28.0 (26.7–32.9)*	*26.8 (23.2–29.0)*	
Tumor Size, *n* (%)			*1.000*
T1	*9 (45.0)*	*9 (45.0)*	
T2	*11 (55.0)*	*11 (55.0)*	
Histological Type, *n* (%)			*1.000*
Invasive Ductal Carcinoma	*17 (85.0)*	*18 (90.0)*	
Invasive Lobular Carcinoma	*3 (15.0)*	*2 (10.0)*	
Molecular Subtype, *n* (%)			*1.000*
Luminal A	*7 (35.0)*	*8 (40.0)*	
Luminal B	*6 (30.0)*	*6 (30.0)*	
HER2-enriched	*4 (20.0)*	*3 (15.0)*	
Triple-Negative	*3 (15.0)*	*3 (15.0)*	
ER Positive, *n* (%)	*13 (65.0)*	*14 (70.0)*	*0.736*
PR Positive, *n* (%)	*12 (60.0)*	*13 (65.0)*	*0.744*
HER2 Positive, *n* (%)	*6 (30.0)*	*5 (25.0)*	*0.723*

*ER* Estrogen receptor, *PR* Progesterone receptor, *HER2* Human epidermal growth factor receptor 2, *IQR* Interquartile range, *SD* Standard deviation

*p*-values calculated using Independent samples t-test (Age, BMI), Chi-square test, or Fisher’s exact test, as appropriate

Pathological examination confirmed balanced tumor biology. Invasive ductal carcinoma was the predominant type (85% ICG vs. 90% PBV, *p* = 1.000). Tumor grade and molecular subtypes (luminal A/B, HER2-enriched, triple-negative) showed no significant differences. Estrogen receptor, progesterone receptor, and HER2/neu status, as well as the Ki-67 proliferation index, were all comparable between groups (all *p* > 0.05).

### Operative outcomes

The type of breast surgery and standard operative parameters were similar between groups. Breast-conserving surgery was performed in 85% of the ICG group and 80% of the PBV group (*p* = 1.000). Total operative time (88.0 ± 9.9 min vs. 87.3 ± 11.3 min, *p* = 0.825) and intraoperative blood loss (66.0 ± 17.1 mL vs. 66.8 ± 19.1 mL, *p* = 0.897) did not differ significantly.

### Sentinel lymph node identification and detection

The performance of the two tracers differed markedly (Table 2). The SLN IR was significantly higher in the ICG group. At least one SLN was identified in all 20 patients (100%) in the ICG group, compared to only 15 patients (75%) in the PBV group (*p* = 0.047).

**Table 2:** Intraoperative sentinel lymph node detection outcomes

Outcome	ICG Group (*n* = 20)	PBV Group (*n* = 20)	*p*-value
SLN Identification Success, *n* (%)	*20 (100)*	*15 (75)*	***0.047***
Detection Time, min			
Mean ± SD	*14.5 ± 2.4*	*24.1 ± 3.0*	***< 0.001***
Median (IQR)	*14.5 (13.0–16.5)*	*24.0 (22.5–26.0)*	
No. of SLNs Detected (Intraop)			
Mean ± SD	*3.0 ± 0.7*	*1.9 ± 0.5*	***< 0.001***
Median (IQR)	*3.0 (2.5–3.5)*	*2.0 (2.0–2.0)*	
No. of SLNs Retrieved (Pathology)			
Mean ± SD	*3.6 ± 1.1*	*2.4 ± 0.9*	***0.002***
Median (IQR)	*3.0 (3.0–4.0)*	*2.0 (2.0–3.0)*	

*IQR *Interquartile range,* SD *Standard deviation

*p*-values calculated using Chi-square test (Identification Success) and Mann–Whitney U test (Detection Time, Node Count)

Under the tracer-specific study protocols, the median time from injection to visualization of the first SLN (detection time) was almost ten minutes shorter in the ICG group than in the PBV group [14.5 min (IQR 13.0–16.5) versus 24.0 min (IQR 22.5–26.0), (*p* < 0.001)].

Furthermore, ICG identified a significantly higher mean number of SLNs per patient intraoperatively. The mean number of SLNs visualized by the surgeon was 3.0 ± 0.7 in the ICG group compared to 1.9 ± 0.5 in the PBV group (*p* < 0.001). A similar finding was observed on final pathological examination, where the mean number of SLNs retrieved per patient was 3.6 ± 1.1 in the ICG group versus 2.4 ± 0.9 in the PBV group (*p* = 0.002).

### Diagnostic accuracy

The diagnostic performance of SLNB was evaluated in all 20 patients in each group using the combined pathological status of the excised SLN(s), when identified, together with the completion ALND specimen as the pathological reference standard (Table 3).

**Table 3:** Mapping outcome and diagnostic performance of sentinel lymph node biopsy

Panel A. Mapping outcome according to final pathological reference standard		
Group / Mapping Outcome	Node-positive	Node-negative
ICG: SLN positive	*8*	*0*
ICG: SLN negative	*0*	*12*
ICG: Mapping failure	*0*	*0*
PBV: SLN positive	*6*	*0*
PBV: SLN negative	*0*	*9*
PBV: Mapping failure	*2*	*3*

Then Panel B:			
Parameter	ICG, all patients	PBV, all patients*	PBV, successful mapping only†
Sensitivity	*100%*	*75.0%*	*100%*
Specificity	*100%*	*100%*	*100%*
PPV	*100%*	*100%*	*100%*
NPV	*100%*	*85.7%*	*100%*
Overall accuracy	*100%*	*90.0%*	*100%*
FNR	*0%*	*25.0%*	*0%*

*All-randomized-patient analysis: the five PBV mapping failures were retained according to the final pathological reference standard; two node-positive mapping failures contributed to the false-negative count and three node-negative mapping failures contributed to the true-negative count

†Sensitivity analysis restricted to the 15 PBV patients in whom an SLN was successfully identified. These estimates describe diagnostic concordance conditional on successful mapping and should be interpreted together with the PBV identification rate of 75%

In the ICG group, SLNB achieved an overall diagnostic accuracy of 100%. It correctly identified all 8 patients with nodal metastases (100% sensitivity) and all 12 patients without metastases (100% specificity), resulting in an NPV of 100% and an FNR of 0%.

In the PBV group, SLN mapping failed in 5 of 20 patients (25%). Of these mapping failures, 2 patients had metastatic axillary LNs on final pathology and 3 had node-negative final pathology. Mapping failures are therefore reported separately from true-negative SLNB results.

In the all-randomized-patient analysis, the two node-positive mapping failures contributed to the false-negative count and the three node-negative mapping failures contributed to the true-negative count according to the final pathological reference standard. Under this analytical approach, PBV yielded a sensitivity of 75%, an NPV of 85.7%, an FNR of 25%, and an overall diagnostic accuracy of 90%.

A sensitivity analysis restricted to the 15 patients with successful PBV mapping showed concordance between SLNB and the final pathological reference standard in all cases: all 6 node-positive patients had a positive SLN and all 9 node-negative patients had a negative SLN. Accordingly, among successfully mapped patients, sensitivity, specificity, NPV, and overall diagnostic accuracy were 100%, with an FNR of 0%. These conditional estimates should be interpreted together with the 25% PBV mapping-failure rate.

### Correlation and safety outcomes

To assess whether the number of metastatic SLNs reflected the total metastatic nodal burden, a paired comparison between the number of positive SLNs and the total number of positive axillary LNs was performed using the Wilcoxon signed-rank test. The test showed no significant paired difference between the number of positive SLNs and the total number of positive axillary LNs in the ICG group (Z = 0.000, *p* = 1.000), whereas a non-significant trend toward fewer positive SLNs than total positive axillary LNs was observed in the PBV group (Z = 1.414, *p* = 0.157).

Tracer-related adverse events were observed exclusively in the PBV group (Table 4). Breast skin discoloration occurred in 15 patients (75%), which gradually faded within two weeks and had completely resolved in 11 patients at the one-month follow-up visit, while 4 patients had residual skin discoloration beyond one month; however, longer follow-up was not performed. Transient urine discoloration occurred in 3 patients (15%) that resolved within the first postoperative day. One patient (5%) experienced a mild allergic skin rash immediately following PBV injection and was treated by intravenous hydrocortisone with no further sequelae. No adverse events related to ICG were reported.

**Table 4:** Tracer-related and postoperative adverse events

Event, *n* (%)	ICG Group (*n* = 20)	PBV Group (*n* = 20)	*p*-value
Tracer-Related			
Any Tracer Event	*0 (0)*	*15 (75)*	***< 0.001***
- Skin Discoloration	*0 (0)*	*15 (75)*	
- Urine Discoloration	*0 (0)*	*3 (15)*	
- Allergic Reaction	*0 (0)*	*1 (5)*	
Postoperative Surgical			
Seroma (Drain Duration, days)	*7.8 ± 2.4*	*8.3 ± 2.3*	*0.445*
Wound Infection	*2 (10)*	*2 (10)*	*1.000*
Wound Dehiscence	*1 (5)*	*1 (5)*	*1.000*
Postoperative Bleeding	*0 (0)*	*0 (0)*	*1.000*

*p*-values calculated using Chi-square/Fisher’s Exact test for categorical variables and Mann–Whitney U test for drain duration

There were no significant differences in postoperative surgical complications between groups. The rate of wound infection (10% in each group) and drain duration (7.8 ± 2.4 days vs. 8.3 ± 2.3 days, *p* = 0.445) were comparable.

## Discussion

SLNB remains a cornerstone of axillary staging in early breast cancer [26, 27], yet the quest for the ideal tracer continues [18, 28, 29]. While blue dyes like PBV have been used for decades, their limitations—including variable IR, allergic potential, and cosmetic drawbacks—are well-documented [30–32]. ICG fluorescence imaging has emerged as a promising alternative, offering real-time, high-contrast visualization of lymphatic channels. However, although several prospective studies and randomized trials have demonstrated promising results (Table 5), evidence directly comparing ICG with blue dye alone remains limited, particularly in resource-limited settings. The present RCT compared the efficacy and safety of ICG fluorescence with PBV as single tracers for SLNB in patients with early-stage breast cancer.

**Table 5:** Representative single-arm and comparative studies evaluating indocyanine green-guided sentinel lymph node biopsy in early breast cancer

Study	Country	Design / Sample size	Tracer	Identification rate
Kitai et al., 2005 [33]	*Japan*	*Original study;* *18 patients*	*ICG*	*94%*
Murawa et al., 2009 [34]	*Germany*	*Prospective study;* *30 patients*	*Initial*: *ICG in 10 patients;* *Then* *ICG + RI in 20 patients*	*Overall*: • *ICG: 97%* *Subgroup*: • *ICG: 100%*, • *RI: 85%*
Hirche et al., 2010 [35]	*Germany*	*Prospective study;* *43 patients*	*ICG*	*97.7%*
Sugie et al., 2010 [36]	*Japan*	*Retrospective multicenter study;* *411 patients*	*ICG + BD*	*ICG: 99%*, *BD: 83%-93%*
Aoyama et al., 2011 [37]	*Japan*	*Prospective study;* *312 patients*	*ICG*	*100%*
Abe et al., 2011 [38]	*Japan*	*Prospective study;* *128 patients*	*ICG + BD*	*ICG: 100%*, *BD: 66%*
Wishart et al., 2012 [39]	*UK*	*Prospective study;* *99 patients*,* 104 procedures*	*ICG + PB + RI*	*ICG: 100%*, *PB: 99%*, *RI: 91.3%*
Hirano et al., 2012 [40]	*Japan*	*Retrospective study;* *501 patients*	*BD vs. BD + ICG*	*BD: 95.7%*, *Dual: 100%*
Sugie et al., 2013 [19]	*Japan*	*Prospective multicenter study;* *99 patients*	*ICG vs. BD*	*ICG: 99%*, *BD: 78%*
Guo et al., 2014 [41]	*China*	*Prospective study;* *86 patients*	*ICG vs. PB vs. ICG + PB*	*ICG: 93%*, *PB: 81.4%*, *Dual: 98.8%*
Tong et al., 2014 [42]	*China*	*Prospective study; 169 patients*	*ICG + PB vs. PB*	*Dual: 96.9%*, *PB: 84.9%*
He et al., 2016 [43]	*China*	*Multicenter prospective study;* *99 patients*	*ICG vs. MB*	*ICG: 99%*, *MB: 91.92%*
Guo et al., 2017 [44]	*China*	*Prospective self-controlled study;* *198 patients*,* 200 procedures*	*ICG + MB*	*ICG: 97%*, *MB: 89%*, *Dual: 99.5%*
Liu et al., 2017 [45]	*China*	*Retrospective study;* *60 patients*	*ICG + MB*	*ICG: 100%*, *MB: 88.3%*
Shen et al., 2018 [46]	*China*	*Prospective study;* *523 patients*	*ICG + MB vs. MB*	*Dual: 99.2%*, *MB: 93.3%*
Qin et al., 2019 [47]	*China*	*Prospective study;* *180 patients*	*ICG + MB vs. MB vs. CN*	*ICG + MB: 100%*, *MB: 96.7%*, *CN: 98.3%*
Wang et al., 2020 [48]	*China*	*Prospective study;* *70 patients*	*ICG vs. MB*	*ICG: 100%*, *MB: 93%*
Lin et al., 2020 [49]	*Taiwan*	*Prospective self-controlled study;* *39 patients*,* 40 procedures*	*ICG + BD*	*ICG: 97.5%*, *BD: 70%*
Hua et al., 2022 [50]	*China*	*Retrospective study;* *194 patients*	*Initial*: *ICG + MB + RI in 44 patients;* *Then* *ICG + MB in 150 patients*	*Overall*: • *ICG: 99%*, • *MB: 92.8%*, • *Dual: 99%* *Triad*: • *ICG: 95.5%*, • *MB: 86.4%*, • *RI: 95.5%*, • *Triad: 100%*
Coibion et al., 2022 [51]	*Belgium*	*Prospective study;* *240 patients*	*ICG vs. PB*	*ICG: 100%*, *PB: 97.5%*
Xu et al., 2022 [52]	*China*	*Retrospective study;* *156 patients*	*ICG + MB vs. ICG vs. MB*	*Dual: 97.4%*, *ICG: 92.9%*, *MB: 84.6%*
Yang et al., 2023 [53]	*China*	*Retrospective study;* *1574 patients*	*ICG + MB*	*99.7%*
da Silva Sá et al., 2023 [54]	*Brazil*	*Prospective study;* *99 patients*	*PB vs. ICG vs. PB + ICG*	*PB: 78.8%*, *ICG: 93.9%*, *Dual: 100%*
Nguyen et al., 2023 [55]	*Australia*	*Comparative study;* *300 patients*	*ICG + RI vs. BD + RI*	*100% in both*
Hounschell et al., 2024 [56]	*USA*	*Retrospective study;* *278 patients*	*RI + BD vs. RI + ICG*	*BD: 97.2%*, *ICG: 98.4%*
Hartmann et al., 2024 [57]	*Germany*	*Prospective study;* *99 patients*,* 100 procedures*	*ICG*	*98.0%*
Pitsinis et al., 2024 / INFLUENCE trial [24]	*UK*	*Prospective study;* *97 patients*	*ICG vs. ICG + RI vs. ICG + PB*	*ICG: 97.9%*, *ICG + RI: 100%*, *ICG + PB: 92%*
Meng et al., 2025 [58]	*China*	*Prospective self-controlled study;* *178 patients*,* 179 procedures*	*MB vs. MB + ICG*	*MB: 92.2%*, *Dual: 93.9%*
Mendoza et al., 2026 [59]	*Peru*	*Retrospective study;* *69 patients*	*ICG*	*100%*

*ICG* Indocyanine Green, *RI* Radioisotope, *BD* Blue Dye, *PB* Patent Blue, *MB* Methylene Blue, *CN* Carbon Nanoparticles

In this trial, the diagnostic performance was assessed using completion ALND following SLNB in all patients. This approach, which has also been utilized in previous validation studies [60, 61], provided a definitive pathological reference standard for calculation of sensitivity, specificity, NPV, and FNR. Although completion ALND is no longer routinely indicated for many patients with early breast cancer, its use in the present study was solely for methodological validation of the mapping techniques rather than for therapeutic purposes.

The intraoperative performance observed in this study appears consistent with trends reported in the broader literature. The IR of 100% with ICG aligns with high success rates documented in other trials, such as the 100% rate reported by Coibion et al. [51] and the near-perfect rates confirmed in a meta-analysis by Goonawardena et al. [62]. The IR of 75% for PBV observed here, while consistent with the lower end of reported ranges, serves to underscore the variability that can be associated with blue dye techniques, even within a protocol-driven setting. The shorter detection time and retrieval of a greater number of SLNs with ICG are also findings that mirror advantages described in other studies, such as those by Hua et al. [50] and da Silva Sá et al. [54].

Our findings are further supported by the recently published multicenter randomized INFLUENCE Trial, which provides the highest level of contemporary evidence evaluating ICG as a standalone tracer for SLNB. In that study, ICG alone achieved an SLN IR of 97.9% and was demonstrated to be non-inferior to ICG combined with either a radioisotope or blue dye, with no significant differences in procedural node positivity rates or overall performance parameters [24]. Similarly, ICG achieved a 100% IR in the present study together with high diagnostic performance. More recently, Pitsinis et al. reported a prospective UK cohort including 300 consecutive SLNB procedures using ICG as the sole tracer. They demonstrated a procedural detection rate of 99.7% and an ICG nodal detection rate of 97.7%, with consistent performance across patient age, body mass index, tumor size, and surgical procedure, further supporting the reliability and reproducibility of ICG-only SLNB in routine clinical practice [25]. Taken together, these contemporary prospective studies substantially strengthen the evidence supporting ICG as a standalone tracer. However, whereas the INFLUENCE Trial [24] evaluated whether a second tracer could be safely omitted and the study by Pitsinis et al. [25] represented a large prospective implementation cohort of ICG-only SLNB, our pilot randomized trial directly compared ICG with PBV as single tracers and evaluated diagnostic performance using completion ALND as the pathological reference standard. Thus, our findings complement the growing body of prospective evidence by providing randomized comparative data demonstrating a higher IR, lower FNR, shorter detection time, and fewer tracer-related adverse events with ICG than with PBV alone. These findings may be particularly relevant in resource-limited settings where radioisotope mapping is unavailable and PBV remains the most commonly used single tracer.

Beyond the higher IR, ICG also demonstrated superior mapping efficiency. The reduction in mean detection time by nearly 10 min (14.45 vs. 24.07 min, *p* < 0.001) is a consistent finding, with da Silva Sá et al. similarly reporting a significantly shorter detection time for ICG compared to blue dye (8.6 vs. 20.6 min) [54]. Furthermore, ICG identified a higher mean number of SLNs both intraoperatively (3.0 vs. 1.87, *p* < 0.001) and on final pathology (3.6 vs. 2.4, *p* = 0.002), consistent with the findings of Coibion et al., who also reported a higher mean SLN yield with ICG (3.6 vs. 3.0) [51]. However, this finding should be interpreted cautiously. A higher nodal yield does not necessarily indicate superior lymphatic mapping and may reflect either improved identification of relevant SLNs or reduced selectivity resulting from tracer diffusion into second-echelon nodes if fluorescence imaging is delayed. Therefore, appropriate timing of ICG imaging is essential to optimize the balance between mapping sensitivity and selectivity.

The favorable diagnostic performance achieved with ICG may be related to its high identification rate together with consistent retrieval of an adequate number of sentinel lymph nodes. The critical finding of a 0% FNR for ICG versus 25% for PBV is particularly notable when contextualized with the literature. While the 25% FNR for PBV in our cohort is higher than some reports, it underscores the potential for significant variability. More importantly, the 0% FNR for ICG aligns with the excellent performance reported in other trials. For instance, Hua et al. also reported a 0% FNR for ICG compared to 8.1% for blue dye [50], and Lin et al. demonstrated a starkly lower FNR for ICG (2.5%) than for blue dye (30%) [49].

The 100% sensitivity and overall accuracy achieved with ICG in our cohort further support its reliability as a standalone tracer, although these findings should be interpreted in light of the relatively small sample size.

Finally, the divergent safety profiles of the two tracers are well-documented and were clearly evident in our results. The absence of adverse events with ICG has been a consistent finding in the literature [29] and is in keeping with the favorable safety profile reported in the recent multicenter randomized INFLUENCE Trial [24]. In contrast, the transient skin discoloration (75%) and allergic reaction rate (5%) associated with PBV in our study are within the spectrum of known complications, which range from cosmetic nuisance to life-threatening anaphylaxis, as detailed in reports by Wilke et al. and Zakaria et al. [30–32].

The potential advantages of ICG may be particularly relevant in institutions where radioisotope mapping is unavailable. Nevertheless, fluorescence-guided SLNB requires dedicated NIR imaging equipment and therefore an initial capital investment. Once this infrastructure is established, however, ICG offers several practical advantages over radioisotope-based mapping, including the absence of ionizing radiation, elimination of nuclear medicine facilities and their associated regulatory requirements, simplified logistics, and avoidance of the recurring costs associated with radioisotope procurement, handling, and disposal. Consequently, although implementation depends on local infrastructure and available resources, ICG may represent a practical alternative with the potential to be cost-effective for centers without access to radioisotope mapping. Formal cost-effectiveness studies are warranted to further evaluate this issue.

## Limitations and future directions

We acknowledge several limitations of this study. First, this was a single-center pilot RCT with a relatively small sample size. Consequently, the study was not designed to provide definitive comparative estimates, and the excellent diagnostic performance observed with ICG, including the 100% IR and absence of false-negative cases, should therefore be interpreted with appropriate caution. Second, blinding of the operating surgeons was not feasible because of the nature of the interventions, although the use of objective pathological endpoints minimized the potential for assessment bias. Finally, the study compared two single-tracer techniques and did not include the current dual-tracer reference standard (radioisotope plus blue dye), reflecting the unavailability of radioisotope mapping at our institution. While the multicenter randomized INFLUENCE Trial supports the use of ICG as a standalone tracer, larger multicenter studies directly comparing ICG alone with the dual-tracer technique are still warranted to further validate these findings.

We also acknowledge that completion level I–II ALND was performed in all participants solely to establish the pathological reference standard required for this diagnostic accuracy study. Although this design was prospectively approved by the institutional Research Ethics Committee and participants provided specific written informed consent, it exposed patients to additional surgery that would not routinely be required under contemporary de-escalation strategies. Furthermore, long-term ALND-related morbidity, including lymphoedema, sensory disturbance, and shoulder dysfunction, was not prospectively collected.

## Final conclusion

In conclusion, in this small single-center randomized controlled trial, ICG fluorescence was associated with a higher SLN identification rate, favorable diagnostic performance, shorter protocol-defined detection time, greater SLN yield, and fewer tracer-related adverse events than PBV as a single tracer for SLNB in patients with clinically node-negative early breast cancer. These preliminary findings add randomized comparative evidence supporting ICG as an effective standalone tracer, particularly in resource-limited settings where radioisotope mapping is unavailable. However, larger multicenter studies comparing ICG alone with the current dual-tracer reference standard, together with formal cost-effectiveness analyses, are needed before widespread adoption can be recommended.

## Data Availability

The datasets generated and/or analyzed during the current study are not publicly available due to patient confidentiality and institutional ethical restrictions. The data are, however, available from the corresponding author on reasonable request and with permission of the Research Ethics Committee of the General Surgery Department, Faculty of Medicine, Ain Shams University.

## References

[1] Kim J, Harper A, McCormack V, Sung H, Houssami N, Morgan E, et al. Global patterns and trends in breast cancer incidence and mortality across 185 countries. Nat Med. 2025;31(4):1154–62. 10.1038/s41591-025-03502-3. 39994475

[2] Bray F, Laversanne M, Sung H, Ferlay J, Siegel RL, Soerjomataram I, et al. Global cancer statistics 2022: GLOBOCAN estimates of incidence and mortality worldwide for 36 cancers in 185 countries. CA Cancer J Clin. 2024;74(3):229–63. 10.3322/caac.21834. 38572751

[3] Lyman GH, Somerfield MR, Bosserman LD, Perkins CL, Weaver DL, Giuliano AE. Sentinel lymph node biopsy for patients with early-stage breast cancer: American Society of Clinical Oncology clinical practice guideline update. J Clin Oncol. 2017;35(5):561–4. 10.1200/JCO.2016.71.0947. 27937089

[4] Veronesi U, Paganelli G, Viale G, Luini A, Zurrida S, Galimberti V, et al. A randomized comparison of sentinel-node biopsy with routine axillary dissection in breast cancer. N Engl J Med. 2003;349(6):546–53. 10.1056/NEJMoa012782. 12904519

[5] Veronesi U, Paganelli G, Viale G, Luini A, Zurrida S, Galimberti V, et al. Sentinel-lymph-node biopsy as a staging procedure in breast cancer: update of a randomised controlled study. Lancet Oncol. 2006;7(12):983–90. 10.1016/S1470-2045(06)70947-0. 17138219

[6] Krag DN, Julian TB, Harlow SP, et al. NSABP-32: phase III, randomized trial comparing axillary resection with sentinel lymph node dissection: a description of the trial. Ann Surg Oncol. 2004;11(Suppl 3):S208–10. 10.1007/BF02523630. 15023753

[7] Land SR, Kopec JA, Julian TB, Brown AM, Anderson SJ, Krag DN, et al. Patient-reported outcomes in sentinel node-negative adjuvant breast cancer patients receiving sentinel-node biopsy or axillary dissection: National Surgical Adjuvant Breast and Bowel Project phase III protocol B-32. J Clin Oncol. 2010;28(25):3929–36. 10.1200/JCO.2010.28.2491. 20679600 PMC2940391

[8] Ashikaga T, Krag DN, Land SR, Julian TB, Anderson SJ, Brown AM, et al. Morbidity results from the NSABP B-32 trial comparing sentinel lymph node dissection versus axillary dissection. J Surg Oncol. 2010;102(2):111–8. 10.1002/jso.21535. 20648579 PMC3072246

[9] Giuliano AE, McCall L, Beitsch P, Whitworth PW, Blumencranz P, Leitch AM et al. Locoregional recurrence after sentinel lymph node dissection with or without axillary dissection in patients with sentinel lymph node metastases: the American College of Surgeons Oncology Group Z0011 randomized trial. Ann Surg. 2010;252(3):426–432;discussion 432–433. 10.1097/SLA.0b013e3181f08f32. PMC5593421 20739842

[10] Giuliano AE, Hunt KK, Ballman KV, Beitsch PD, Whitworth PW, Blumencranz PW, et al. Axillary dissection vs no axillary dissection in women with invasive breast cancer and sentinel node metastasis: a randomized clinical trial. JAMA. 2011;305(6):569–75. 10.1001/jama.2011.90. 21304082 PMC5389857

[11] Giuliano AE, Ballman K, McCall L, Beitsch P, Whitworth PW, Blumencranz P, et al. Locoregional recurrence after sentinel lymph node dissection with or without axillary dissection in patients with sentinel lymph node metastases: long-term follow-up from the American College of Surgeons Oncology Group (Alliance) ACOSOG Z0011 randomized trial. Ann Surg. 2016;264(3):413–20. 10.1097/SLA.0000000000001863. 27513155 PMC5070540

[12] de Boniface J, Filtenborg Tvedskov T, Rydén L, Szulkin R, Reimer T, Kühn T, et al. Omitting axillary dissection in breast cancer with sentinel-node metastases. N Engl J Med. 2024;390(13):1163–75. 10.1056/NEJMoa2313487. 38598571

[13] Donker M, van Tienhoven G, Straver ME, Meijnen P, van de Velde CJ, Mansel RE, et al. Radiotherapy or surgery of the axilla after a positive sentinel node in breast cancer (EORTC 10981–22023 AMAROS): a randomised, multicentre, open-label, phase 3 non-inferiority trial. Lancet Oncol. 2014;15(12):1303–10. 10.1016/S1470-2045(14)70460-7. 25439688 PMC4291166

[14] Bartels SAL, Donker M, Poncet C, Sauvé N, Straver ME, van de Velde CJH, et al. Radiotherapy or surgery of the axilla after a positive sentinel node in breast cancer: 10-year results of the randomized controlled EORTC 10981–22023 AMAROS trial. J Clin Oncol. 2023;41(12):2159–65. 10.1200/JCO.22.01565. 36383926

[15] Sávolt Á, Péley G, Polgár C, Udvarhelyi N, Rubovszky G, Kovács E, et al. Eight-year follow-up result of the OTOASOR trial: the Optimal Treatment of the Axilla—Surgery or Radiotherapy after positive sentinel lymph node biopsy in early-stage breast cancer: a randomized, single-centre, phase III, non-inferiority trial. Eur J Surg Oncol. 2017;43(4):672–9. 10.1016/j.ejso.2016.12.011. 28139362

[16] Gentilini OD, Botteri E, Sangalli C, Galimberti V, Porpiglia M, Agresti R, et al. Sentinel lymph node biopsy vs no axillary surgery in patients with small breast cancer and negative results on ultrasonography of axillary lymph nodes: the SOUND randomized clinical trial. JAMA Oncol. 2023;9(11):1557–64. 10.1001/jamaoncol.2023.3759. 37733364 PMC10514873

[17] Reimer T, Stachs A, Veselinovic K, Kühn T, Heil J, Polata S, et al. Axillary surgery in breast cancer: primary results of the INSEMA trial. N Engl J Med. 2025;392(11):1051–64. 10.1056/NEJMoa2412063. 39665649

[18] Linehan DC, Hill AD, Akhurst T, Yeung H, Yeh SD, Tran KN, et al. Intradermal radiocolloid and intraparenchymal blue dye injection optimize sentinel node identification in breast cancer patients. Ann Surg Oncol. 1999;6(5):450–4. 10.1007/s10434-999-0450-4. 10458682

[19] Sugie T, Sawada T, Tagaya N, et al. Comparison of the indocyanine green fluorescence and blue dye methods in detection of sentinel lymph nodes in early-stage breast cancer. Ann Surg Oncol. 2013;20(7):2213–8. 10.1245/s10434-013-2890-0. 23429938

[20] Reyes FJ, Noelck MB, Valentino C, Grasso-LeBeau L, Lang JE. Complications of methylene blue dye in breast surgery: case reports and review of the literature. J Cancer. 2011;2:20–5. 10.7150/jca.2.20. PMC3005551 21197261

[21] Alander JT, Kaartinen I, Laakso A, Pätilä T, Spillmann T, Tuchin VV, et al. A review of indocyanine green fluorescent imaging in surgery. Int J Biomed Imaging. 2012;2012:940585. 10.1155/2012/940585. 22577366 PMC3346977

[22] Abd-erRazik MA, Elghandour AM, Osman A, Abdel Hamid MAS. Indocyanine green fluorescent cholangiography and intraoperative angiography with laparoscopic cholecystectomy: a randomized controlled trial. Egypt J Surg. 2022;41(1):153–60. 10.4103/ejs.ejs_302_21.

[23] Hope-Ross M, Yannuzzi LA, Gragoudas ES, Guyer DR, Slakter JS, Sorenson JA, et al. Adverse reactions due to indocyanine green. Ophthalmology. 1994;101(3):529–33. 10.1016/S0161-6420(94)31303-0. 8127574

[24] Pitsinis V, Kanitkar R, Vinci A, Choong WL, Benson J. Results of a prospective randomized multicenter study comparing indocyanine green fluorescence combined with a standard tracer versus indocyanine green alone for sentinel lymph node biopsy in early breast cancer: the INFLUENCE trial. Ann Surg Oncol. 2024;31(13):8848–55. 10.1245/s10434-024-16176-x. 39266795 PMC11549146

[25] Pitsinis V, Khitab N, Jordan L, Vinci A, Kanitkar R, Elseedawy E, et al. ICG-only sentinel lymph node biopsy in breast cancer: a prospective UK cohort demonstrating consistent detection across patient and tumour characteristics—a follow-on to the INFLUENCE trial. Ann Surg Oncol. 2026. 10.1245/s10434-026-20316-w. 42527803 PMC13675259

[26] Nieweg OE, Uren RF, Thompson JF. The history of sentinel lymph node biopsy. Cancer J. 2015;21(1):3–6. 10.1097/PPO.0000000000000091. 25611772

[27] Bergkvist L, Frisell J. Management of the axilla: sentinel lymph node biopsy. In: Wyld L, Markopoulos C, Leidenius M, Senkus-Konefka E, editors. Breast cancer management for surgeons: a European multidisciplinary textbook. Cham: Springer; 2018. pp. 275–84. 10.1007/978-3-319-56673-3_23.

[28] Kuehn T, Vogl FD, Helms G, Pueckler SV, Schirrmeister H, Strueber R, et al. Sentinel-node biopsy for axillary staging in breast cancer: results from a large prospective German multi-institutional trial. Eur J Surg Oncol. 2004;30(3):252–9. 10.1016/j.ejso.2003.11.016. 15028305

[29] Abd-erRazik MA, Rashed AM. Indocyanine green fluorescence-guided sentinel node biopsy during surgery for breast cancer. Ain Shams J Surg. 2023;16(1):73–5. 10.21608/asjs.2023.285752.

[30] Aydogan F, Celik V, Uras C, Salihoglu Z, Topuz U. A comparison of the adverse reactions associated with isosulfan blue versus methylene blue dye in sentinel lymph node biopsy for breast cancer. Am J Surg. 2008;195(2):277–8. 10.1016/j.amjsurg.2007.03.008. 18194680

[31] Wilke LG, McCall LM, Posther KE, Whitworth PW, Reintgen DS, Leitch AM, et al. Surgical complications associated with sentinel lymph node biopsy: results from a prospective international cooperative group trial. Ann Surg Oncol. 2006;13(4):491–500. 10.1245/ASO.2006.05.013. 16514477

[32] Zakaria S, Hoskin TL, Degnim AC. Safety and technical success of methylene blue dye for lymphatic mapping in breast cancer. Am J Surg. 2008;196(2):228–33. 10.1016/j.amjsurg.2007.08.060. 18367146

[33] Kitai T, Inomoto T, Miwa M, Shikayama T. Fluorescence navigation with indocyanine green for detecting sentinel lymph nodes in breast cancer. Breast Cancer. 2005;12(3):211–5. 10.2325/jbcs.12.211. 16110291

[34] Murawa D, Hirche C, Dresel S, Hünerbein M. Sentinel lymph node biopsy in breast cancer guided by indocyanine green fluorescence. Br J Surg. 2009;96(11):1289–94. 10.1002/bjs.6721. 19847873

[35] Hirche C, Murawa D, Mohr Z, Kneif S, Hünerbein M. ICG fluorescence-guided sentinel node biopsy for axillary nodal staging in breast cancer. Breast Cancer Res Treat. 2010;121(2):373–8. 10.1007/s10549-010-0760-z. 20140704

[36] Sugie T, Kassim KA, Takeuchi M, Hashimoto T, Yamagami K, Masai Y, et al. A novel method for sentinel lymph node biopsy by indocyanine green fluorescence technique in breast cancer. Cancers (Basel). 2010;2(2):713–20. 10.3390/cancers2020713. 24281090 PMC3835100

[37] Aoyama K, Kamio T, Ohchi T, Nishizawa M, Kameoka S. Sentinel lymph node biopsy for breast cancer patients using fluorescence navigation with indocyanine green. World J Surg Oncol. 2011;9:157. 10.1186/1477-7819-9-157. 22132943 PMC3269998

[38] Abe H, Mori T, Umeda T, Tanaka M, Kawai Y, Shimizu T, et al. Indocyanine green fluorescence imaging system for sentinel lymph node biopsies in early breast cancer patients. Surg Today. 2011;41(2):197–202. 10.1007/s00595-009-4254-8. 21264754

[39] Wishart GC, Loh SW, Jones L, Benson JR. A feasibility study (ICG-10) of indocyanine green fluorescence mapping for sentinel lymph node detection in early breast cancer. Eur J Surg Oncol. 2012;38(8):651–6. 10.1016/j.ejso.2012.05.007. 22704050

[40] Hirano A, Kamimura M, Ogura K, et al. A comparison of indocyanine green fluorescence imaging plus blue dye and blue dye alone for sentinel node navigation surgery in breast cancer patients. Ann Surg Oncol. 2012;19(13):4112–6. 10.1245/s10434-012-2478-0. 22782671

[41] Guo W, Zhang L, Ji J, Gao W, Liu J, Tong M. Evaluation of the benefit of using blue dye in addition to indocyanine green fluorescence for sentinel lymph node biopsy in patients with breast cancer. World J Surg Oncol. 2014;12:290. 10.1186/1477-7819-12-290. 25239029 PMC4182872

[42] Tong M, Guo W, Gao W. Use of fluorescence imaging in combination with patent blue dye versus patent blue dye alone in sentinel lymph node biopsy in breast cancer. J Breast Cancer. 2014;17(3):250–5. 10.4048/jbc.2014.17.3.250. 25320623 PMC4197355

[43] He K, Chi C, Kou D, Huang W, Wu J, Wang Y, et al. Comparison between the indocyanine green fluorescence and blue dye methods for sentinel lymph node biopsy using novel fluorescence image-guided resection equipment in different types of hospitals. Transl Res. 2016;178:74–80. 10.1016/j.trsl.2016.07.010. 27497181

[44] Guo J, Yang H, Wang S, Cao Y, Liu M, Xie F, et al. Comparison of sentinel lymph node biopsy guided by indocyanine green, blue dye, and their combination in breast cancer patients: a prospective cohort study. World J Surg Oncol. 2017;15(1):196. 10.1186/s12957-017-1264-7. 29096643 PMC5667473

[45] Liu J, Huang L, Wang N, Chen P. Indocyanine green detects sentinel lymph nodes in early breast cancer. J Int Med Res. 2017;45(2):514–24. 10.1177/0300060516687149. 28415938 PMC5536661

[46] Shen S, Xu Q, Zhou Y, Mao F, Guan J, Sun Q. Comparison of sentinel lymph node biopsy guided by blue dye with or without indocyanine green in early breast cancer. J Surg Oncol. 2018;117(8):1841–7. 10.1002/jso.25058. 29790178

[47] Qin X, Yang M, Zheng X. Comparative study of indocyanine green combined with blue dye with methylene blue only and carbon nanoparticles only for sentinel lymph node biopsy in breast cancer. Ann Surg Treat Res. 2019;97(1):1–6. 10.4174/astr.2019.97.1.1. 31297346 PMC6609418

[48] Wang Z, Cui Y, Zheng M, Ge H, Huang Y, Peng J, et al. Comparison of indocyanine green fluorescence and methylene blue dye in the detection of sentinel lymph nodes in breast cancer. Gland Surg. 2020;9(5):1495–501. 10.21037/gs-20-671. 33224824 PMC7667080

[49] Lin J, Lin LS, Chen DR, Lin KJ, Wang YF, Chang YJ. Indocyanine green fluorescence method for sentinel lymph node biopsy in breast cancer. Asian J Surg. 2020;43(12):1149–53. 10.1016/j.asjsur.2020.02.003. 32143963

[50] Hua B, Li Y, Yang X, Ren X, Lu X. Short-term and long-term outcomes of indocyanine green for sentinel lymph node biopsy in early-stage breast cancer. World J Surg Oncol. 2022;20(1):253. 10.1186/s12957-022-02719-7. 35941602 PMC9361589

[51] Coibion M, Olivier F, Courtois A, Maes N, Jossa V, Jerusalem G. A randomized prospective non-inferiority trial of sentinel lymph node biopsy in early breast cancer: blue dye compared with indocyanine green fluorescence tracer. Cancers (Basel). 2022;14(4):888. 10.3390/cancers14040888. 35205636 PMC8870473

[52] Xu Y, Yuan S, Chen M, Gong K, Liu Y, Li S, et al. Evaluation of indocyanine green combined with methylene blue staining in sentinel lymph node biopsy of breast cancer. Gland Surg. 2022;11(9):1489–96. 10.21037/gs-22-434. 36221275 PMC9547706

[53] Yang R, Dong C, Jiang T, Zhang X, Zhang F, Fan Z. Indocyanine green and methylene blue dye-guided sentinel lymph node biopsy in early breast cancer: a single-center retrospective survival study in 1574 patients. Clin Breast Cancer. 2023;23(4):408–14. 10.1016/j.clbc.2023.02.002. 36907808

[54] da Silva Sá R, Von Ah Rodrigues RF, Bugalho LA, da Silva SU, Pinto Nazário AC. Evaluation of the efficacy of using indocyanine green associated with fluorescence in sentinel lymph node biopsy. PLoS ONE. 2023;18(10). 10.1371/journal.pone.0273886. PMC10599532 37878619

[55] Nguyen CL, Zhou M, Easwaralingam N, Seah JL, Azimi F, Mak C, et al. Novel dual tracer indocyanine green and radioisotope versus gold standard sentinel lymph node biopsy in breast cancer: the GREENORBLUE trial. Ann Surg Oncol. 2023;30(11):6520–7. 10.1245/s10434-023-13824-6. 37402976 PMC10507001

[56] Hounschell CA, Kilgore LJ, Pruitt P, Wilder C, Balanoff CR, Wagner JL, et al. Evaluation of learning curve with indocyanine green versus blue dye for sentinel lymph node biopsy in breast cancer. Am J Surg. 2024;227:218–23. 10.1016/j.amjsurg.2023.10.003. 37838506

[57] Hartmann S, Plonus ML, Schultek G, Stubert J, Gerber B, Reimer T. Indocyanine green marking of axillary sentinel lymph nodes in early breast cancer. Geburtshilfe Frauenheilkd. 2025;85(6):631–8. 10.1055/a-2436-1699. 40510404 PMC12158541

[58] Meng L, Zhu M, Yuan Y, Li Y, Wu H, Zhang W. Assessing the benefit of using indocyanine green in addition to methylene blue for breast cancer sentinel lymph node biopsy. Med (Baltim). 2025;104(20). 10.1097/MD.0000000000042500. PMC12091630 40388739

[59] De Mendoza GW, Muñoz Quispe NE, Aguilar Cosme JM, Mendoza S, Hidalgo Ramos RE, Díaz-Llontop D, et al. Clinicopathological factors associated with sentinel lymph node positivity in breast cancer using indocyanine green: a retrospective study in Peru. World J Surg Oncol. 2026;24(1):112. 10.1186/s12957-025-04183-5. 41652639 PMC12973559

[60] Fattahi AS, Tavassoli A, Rohbakhshfar O, Sadeghi R, Abdollahi A, Forghani MN. Can methylene blue dye be used as an alternative to patent blue dye to find the sentinel lymph node in breast cancer surgery? J Res Med Sci. 2014;19(10):918–22. 25538772 PMC4274565

[61] Augustine P, Dasu S, Nair SP, Bhargavan RV, Pradeep VM. Validation of sentinel lymph node biopsy technique using dual tracer technique in post-lumpectomy early breast cancer patients. Indian J Surg Oncol. 2023;14(2):434–9. 10.1007/s13193-020-01242-z. 37324305 PMC10267054

[62] Goonawardena J, Yong C, Law M. Use of indocyanine green fluorescence compared with radioisotope for sentinel lymph node biopsy in early-stage breast cancer: a systematic review and meta-analysis. Am J Surg. 2020;220(3):665–76. 10.1016/j.amjsurg.2020.02.001. 32115177

